# The larvicide pyriproxyfen blamed during the Zika virus outbreak does not cause microcephaly in zebrafish embryos

**DOI:** 10.1038/srep40067

**Published:** 2017-01-04

**Authors:** Stefania Dzieciolowska, Anne-Laure Larroque, Elizabeth-Ann Kranjec, Pierre Drapeau, Eric Samarut

**Affiliations:** 1 Research Center of the University of Montreal Hospital Center (CRCHUM), Department of Neurosciences, Université de Montréal, Montréal, QC, Canada; 2 Drug Discovery Platform, Research Institute of the McGill University Health Center, Montréal, QC, Canada; 3 DanioDesign Inc., Montréal, QC, Canada

## Abstract

Although the zika virus (ZIKV) has now been strongly correlated with emerging cases of microcephaly in the Americas, suspicions have been raised regarding the use of pyriproxyfen, a larvicide that prevents mosquito development, in drinking water. The effects of this compound on neurodevelopment have not yet been addressed specifically in vertebrates. As a result, we aimed at addressing the effects, if any, of pyriproxyfen on neurodevelopment in the zebrafish embryo as a vertebrate model. Using zebrafish transgenic lines expressing GFP in different cell populations (*elavl3* in newborn neurons, *gfap* and *nestin* in neural stem cells), we focused on the analysis of whole embryonic brain volume after confocal 3D-reconstruction and the quantification of purified neural stem cells during early neurodevelopment by FACS-cell sorting from whole *in vivo* embryos. Interestingly, though lethal at very high doses, pyriproxyfen did not cause brain malformation nor any significant changes in the number of observed stem cells in the developing central nervous system. Our data indicate that pyriproxyfen does not affect central nervous system development in zebrafish, suggesting that this larvicide on its own, may not be correlated with the increase in microcephaly cases reported recently.

Recently, the incidence of reported microcephaly cases has vastly increased in the Americas since the rapid emergence of the Zika virus (ZIKV), a flavivirus related to dengue, West Nile and yellow fever viruses^1^,^2^,^3^. Since its detection in Brazil in early December 2015, ZIKV has spread extensively where cases of the viral infection have been reported in most countries in Latin America as well as in the Caribbean, and have been reported to be associated with microcephaly^1^,^2^,^4^.

Microcephaly is a rare neurological condition in which an infant or newborn presents with a head circumference that is more than 3 standard deviations (SDs) below the mean of other children of the same age and sex^5^. It is considered a developmental defect of the brain and is a result of improper brain development during pregnancy (congenital) or may be due to a halt of brain growth after birth. Depending on the severity of the microcephaly, affected children may present with developmental delays, difficulty with motor coordination, facial distortions, seizures and mental retardation^6^. This condition can be caused by a variety of genetic and environmental factors such as craniosynostosis, chromosomal abnormalities, decreased oxygen to the fetal brain, exposure to drugs or alcohol during pregnancy, malnutrition, uncontrolled maternal phenylketonuria or diabetes as well as infections of the fetus during pregnancy^7^,^8^,^9^,^10^,^11^,^12^.

Since December 2015, Brazil has reported 440,000 to 1,300,000 local cases of ZIKV, making it one of the most affected countries by the ZIKV epidemic^13^,^14^. Prior to the outbreak, Brazil reported, on average, 150 to 200 cases of microcephaly between 2010 and 2014, whereas by November 2015, 1,248 new cases of microcephaly had been reported, 509 of which were reported between November 21^st^ and 28^th^ 2015^15^. Furthermore, currently available serological tests are unable to reliably distinguish ZIKV from other flaviviruses, hindering a rapid and effective public health response to the epidemic.

It has been suggested that ZIKV was first introduced to Brazil by an infected traveler or mosquito and its subsequent spread across the region was likely facilitated through *Aedes Aegypti*, the principal vector of the virus, among other *Aedes* species of mosquitoes^1^,^13^. Since the recent ZIKV epidemic, there has been a significant increase in the number of microcephaly cases reported in these regions. Many questions remain regarding the transmission of the virus and its role in microcephaly, but studies examining fetuses and infants with microcephaly have shown the presence of ZIKV RNA in the placenta, amniotic fluid and fetal tissues and have also shown the presence of the virus in brain tissues, suggesting it plays a role in the development of this condition^16^,^17^.

Despite the strong association recently discovered between ZIKV and microcephaly, anecdotal arguments have been made that other environmental factors may have lead to the outbreak of microcephaly cases in the Americas. More specifically, claims were made that speculated whether the use of the larvicide pyriproxyfen may have played a role in the emergence of reported microcephaly cases. To date, few studies have examined the effect of this larvicide in relation to neurodevelopment and the central nervous system. Pyriproxyfen is a pyridine-based larvicide categorized as a juvenile hormone analog pesticide that regulates a variety of processes in postembryonic development and adult reproduction in insects, resulting in a disruption in the insect life cycle and the failure of egg hatching in many insect species^18^,^19^,^20^,^21^. Beginning in 2014, pyriproxyfen was used in Brazilian water supplies to fight the proliferation of mosquito larvae and the use of which was in line with the World Health Organization (WHO)’s Guidelines for Drinking water Quality (GDWQ)^22^ and Pesticide Evaluation Scheme (WHOPES)^23^. Despite its popular use in controlling agricultural, household and product pests, pyriproxyfen has continued to be rumoured to be involved in the development of microcephaly by claims in a report from an Argentinian organization called the “Physicians in Crop-Sprayed Towns”^24^ that made conclusions from a previous report from the Brazillian Association of Collective Health^25^. The former report, which received widespread media attention^26^,^27^,^28^, alleged that the pesticide was at fault for the rise in reported microcephaly cases, though no scientific work had been performed to support these claims. As a result of misinformation, some regions of Brazil have suspended the use of pyriproxyfen, creating possibly a more dangerous environment for the virus to spread^29^,^30^.

The metabolism of pyriproxyfen has been exhaustively documented in rats, goats and hens^31^. However, to date, few studies have investigated the effects of pyriproxyfen on animal development and physiology. In one report by the Food and Agriculture Organization (FAO), that assessed developmental neurotoxicity by examining behaviour, motor and sensory responses, it was found that pyriproxyfen caused little developmental toxicity and was not teratogenic in rats and rabbits^32^. Additionally, in 1989, Koyama *et al*. performed an exhaustive blood analysis of rats that were fed with pyriproxyfen-containing pellets^33^. Although they noticed minor changes in very specific blood features, they did not report premature death nor any developmental defects. Surprisingly, since claims against pyriproxyfen have been made, the effects of this compound on the development of the nervous system have not been addressed specifically in vertebrates.

As a result, in this work, we aimed at addressing the effects, if any, of pyriproxyfen on the neurodevelopment of the zebrafish embryo. Indeed, due to its fast development, optical transparency for imaging of labelled cell populations and its relative facility in handling, zebrafish have emerged as a convenient vertebrate model to study toxic effects of compounds in a high-throughput manner^34^. Moreover, the zebrafish embryo can be easily exposed to chemicals by directly treating the bathing water in which the fish are incubated. Thus, using zebrafish embryos as a model could help draw conclusions on the uncertainties surrounding this compound and help guide further research on microcephaly.

## Results

### Pyriproxyfen is lethal at high doses, but does not affect zebrafish embryo development at the maximum recommended dose used in practice

**Figure 1:**
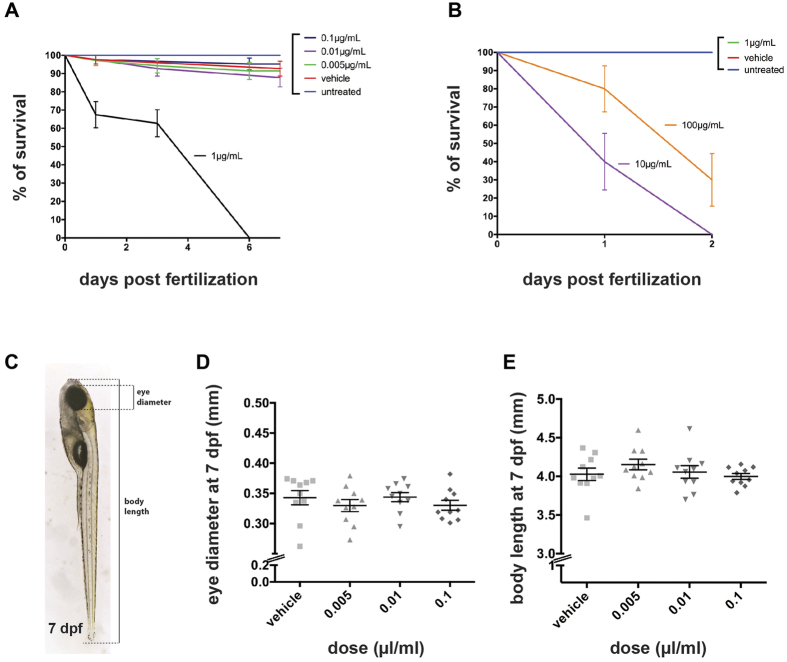
Pyriproxyfen is toxic at high doses. (**A**) Percentage of survival of embryos treated from 2–4 cell stage with various pyriproxyfen concentrations ranging from 0,005 μg/ml to 1 μg/ml. The latest dose appears to be lethal with 100% of death after 5 days of development. Lower doses of pyriproxyfen (0,005; 0,01; 0,1 μg/ml) do not induce a severe decrease in the survival rate (N = 2 batches, n = 20 embryos per condition). (**B**) Higher doses of pyriproxyfen (10 and 100 μg/ml) are lethal as early as 2 dpf (N = 2, n = 20). (**C**) Representation of measurements performed on 7 dpf larvae to assess gross morphology effects. (**D**,**E**) Eye diameter and body length at 7 dpf of pyriproxyfen-treated embryos are not significantly different from vehicle (EtOH)- treated embryos (*p* > 0.05) (n = 10 per condition).

Pyriproxyfen is usually used by treating the drinking water at a maximum final concentration of 0.01 mg/L (0.01 μg/ml) as recommended by the World Health Organization (WHO)^22^. With the aim of performing a rigorous toxicity assay, and as recommended by the Organisation for Economic Co-operation and Development (OECD) in their Fish Embryo Acute Toxicity (FET) Test^35^,^36^, we used five different concentrations surrounding the maximum recommended dose. As a result, we treated freshly laid embryos (from 2–4 cell-stage onwards) with increasing doses of pyriproxyfen from 0.005 μg/ml up to 1 μg/ml. Of note, all the incubations were performed in glass vials to prevent any sequestration of the compound by plastic. After four days post fertilization (dpf), we noticed that the dose of 1 μg/ml led to a decrease of approximately 50% in the survival rate and was fully lethal after 7 dpf (*p* < 0.0001, Logrank test, N = 2, n = 20; Fig. 1A). Of note is that we obtained the same survival results if pyriproxyfen was diluted in DMSO instead of ethanol (Supplementary Figure 1). This observation is consistent with the half maximal effective concentration (EC_50_) of 1.6 μg/ml (i.e. 5 μM) that has been found in a previous study in zebrafish^37^. Consistently, we also treated embryos with extremely high doses of pyriproxyfen, concentrations ranging from 10 to 100 μg/ml, and noticed a high level of toxicity leading to death in 100% of the embryos as early as 2 dpf (*p* < 0.0001, Logrank test, N = 2, n = 20; Fig. 1B).

**Figure 2:**
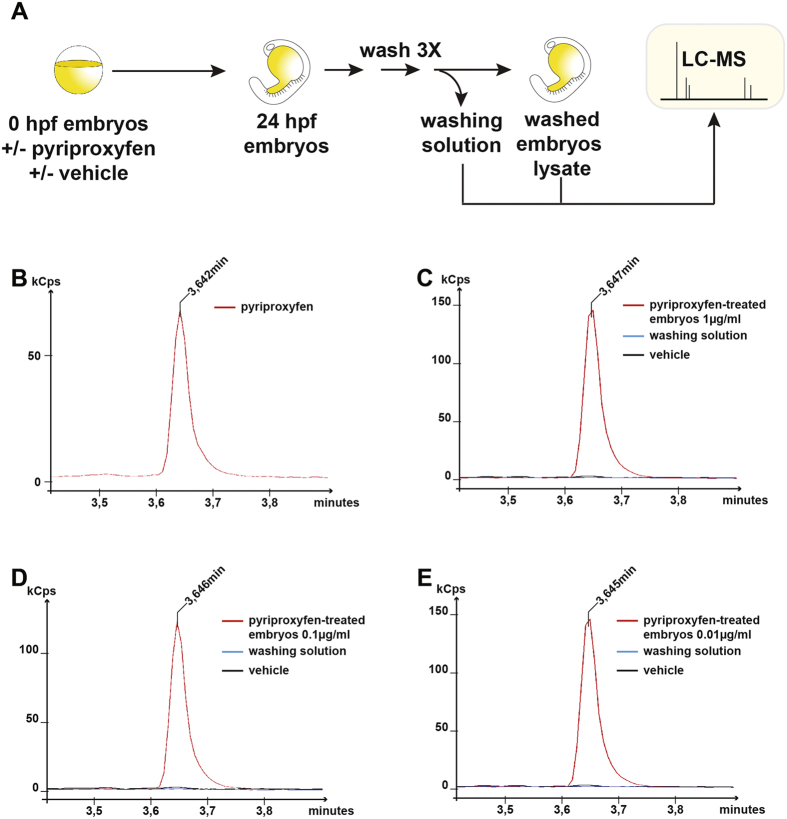
Liquid chromatography-mass spectrometry detection of pyriproxyfen in treated embryos. (**A**) Steps involved in the preparation for pyriproxyfen extraction of embryos (hpf: hours post-fertilization). (**B**) LC chromatogram of commercial pyriproxyfen at a concentration of 1.56 nM eluting at a retention time of 3.64 min. (**C**–**E**) LC chromatogram illustrating pyriproxyfen extraction from embryos treated with different concentrations of the molecule compared to vehicle treated embryos and the 3rd washing solution. (Cps: counts per second).

Although we observed a lethal effect at high dose exposure, which suggests the ability of pyriproxyfen to penetrate the chorion surrounding the embryo, we wanted to ensure that the compound was indeed reaching the embryo even when applied at low concentrations (i.e 0,01 μg/ml). To do so, using liquid chromatography-mass spectrometry (LC-MS), we showed that pyriproxyfen was present in 24-hour embryos treated with different doses of pyriproxyfen (1, 0.1 and 0.01 μg/ml) (Fig. 2). Moreover, we were able to quantify the amount of pyriproxyfen per embryo and showed that this amount is strictly proportional to the concentration applied in the bathing medium (Supplementary Figure 2). These results demonstrate that pyriproxyfen penetrated the chorion and entered the embryonic tissue effectively at all concentrations tested. We also analysed four different concentrations of pyriproxyfen surrounding the maximum dose of pyriproxyfen used for water treatment as recommended by the WHO^22^. As the 10 μg/ml and 100 μg/ml concentrations caused early lethality, we decided to utilize the 1 μg/ml concentration as the highest dose that would be tested further in our toxicity assay.

Next, we decided to analyze the effects of pyriproxyfen on the general morphology of the zebrafish larvae (Fig. 1C–E). To do so, we measured total body length and eye diameter throughout larval development (2 and 7 dpf) (Fig. 1C), features that can reflect evidence of developmental deficits. As shown, all these measurements depicted no significant differences in eye diameter (*p* > 0.05, one-way ANOVA, Dunnett’s multiple comparisons test, n = 10; Fig. 1D) or body length (*p* > 0.05, one-way ANOVA, Dunnett’s multiple comparisons test, n = 10; Fig. 1E) when comparing pyriproxyfen-treated concentrations to vehicle-treated larvae.

Altogether, these data indicate that at moderate doses (up to 100-fold the maximum recommended dose of pyriproxyfen used in practice), pyriproxyfen does not induce abnormalities in the general development of the zebrafish larvae as far as it can be judged morphologically. However, at doses higher than 0.1 μg/ml, pyriproxyfen is lethal in zebrafish embryos.

### Pyriproxyfen does not alter central nervous system development of zebrafish embryo

**Figure 3:**
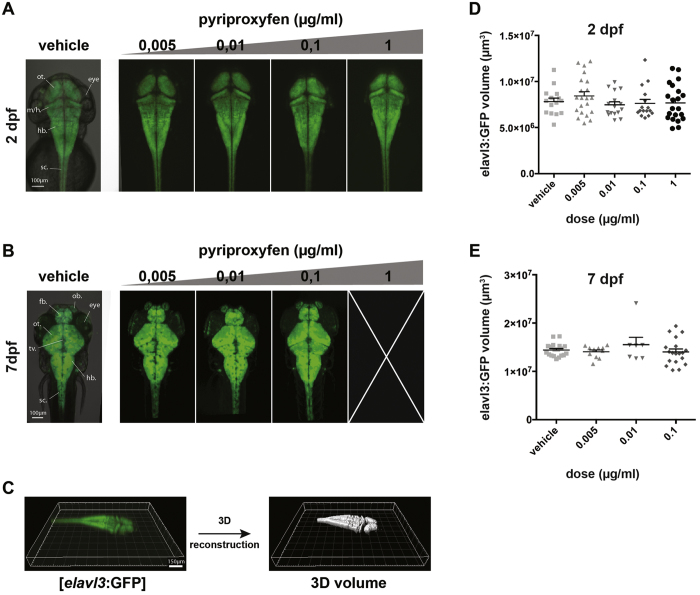
Embryonic and larval brain volumes are not affected by pyriproxyfen treatment. (**A**) Transgenic [*elavl3:*GFP] 2 dpf embryo treated with vehicle, 0,005 μg/ml, 0,01 μg/ml, 0,1 μg/ml and 1 μg/ml pyriproxyfen were imaged under a confocal microscope. The morphology of the embryonic brain is not affected by any of the doses. (**B**) Transgenic [*elavl3:*GFP] 7 dpf larvae treated with vehicle, 0,005 μg/ml, 0,01 μg/ml, 0,1 μg/ml pyriproxyfen were imaged under a confocal microscope. Of note is that none of the embryos treated with a dose of 1 μg/ml survived past 6 dpf. The morphology of the larval brain is not affected by any of the doses. (**C**) 3D-volume reconstruction of [*elavl3:*GFP] embryos using Imaris software (Bitplane) and confocal microscopy. (**D**,**E**) Quantification of embryonic (2 dpf) (**D**) and larval (7 dpf) (**E**) brain volumes shows no significant differences between vehicle-treated and pyriproxyfen-treated embryos (*p* > 0.05). (N = 2, n = 7 per condition). sc.: spinal cord; hb.: hindbrain; m/h: midbrain/hindbrain boundary; mb.: midbrain; fb.: forebrain; ot.: optic tectum; tv.: tectum ventricle; ob.: olfactory bulb.

Although the main morphological features are not affected by the maximum recommended dose of pyriproxyfen used in practice (0.01 μg/ml), we decided to specifically analyze its effects on the development of the central nervous system, in particular on the developing brain. To do so, we took advantage of the [*elavl3:*GFP] transgenic lines^38^ that express GFP in all post-mitotic neurons, that is to say in all developmentally mature nervous structures. Indeed, we treated 2–4 cell stage embryos from this transgenic line with increasing doses of pyriproxyfen and monitored brain morphology at 2 and 7 dpf (Fig. 3). The comparison of the general structure of the embryonic brain did not appear to be significantly affected in any of the pyriproxyfen-treated groups compared to the vehicle-treated group at 2 dpf (Fig. 3A) or at 7 dpf (Fig. 3B).

Although these results suggest no specific effects of pyriproxyfen on the development of the main brain structures, we decided to analyze in more detail whether the embryonic and larval brain volumes were affected following exposure to pyriproxyfen. This assay would help decipher a potential correlation between pyriproxyfen and newborn microcephaly cases, as this condition is a result of reduced brain growth and volume. We imaged embryos in every condition at 2 dpf (embryonic stage) as well as at 7 dpf (larval stage) using confocal microscopy. The depth of the imaging performed ensured that the whole GFP-positive brain structure was taken into account. Next, using Imaris (Bitplane) automated software, we were able to reconstruct a 3D-output of the brain nervous matter defined by GFP expression from [*elavl3:*GFP] transgenic embryos (Fig. 3C).

In order to ensure that our brain calculation method is quantitative enough to detect differences in brain volumes, we injected a morpholino against *clpb* that has been previously described to induce microcephaly in zebrafish^39^ (Supplementary Figure 3). Using our method, we found that the brain volume of morpholino-injected embryos at 2 dpf was significantly reduced by about 30% compared to non-injected embryos (Supplementary Figure 3). As a result, this data ensures that our method is sensitive enough to detect a putative effect of pyriproxyfen on larval brain volume. After calculating the embryonic (2 dpf) and larval (7 dpf) brain volumes, we were not able to identify any significant differences among the various doses tested both at 2 dpf (*p* > 0.05, one-way ANOVA, Dunnett’s multiple comparisons test, N = 2, n = 7; Fig. 3D) nor at 7 dpf (*p* > 0.05, Kruskal-Wallis, Dunn’s multiple comparisons test, N = 2, n = 7; Fig. 3E), when compared to vehicle-treated embryos/larvae. As we did not notice any differences at either developmental stage, this suggests that neither the embryonic developing brain nor the more mature larval brain was morphologically affected by pyriproxyfen exposure.

Altogether, these data suggests that pyriproxyfen exposure does not affect brain structure or brain nervous tissue volume in the developing zebrafish larvae.

### The main neural stem cell populations are not affected by pyriproxyfen exposure

**Figure 4:**
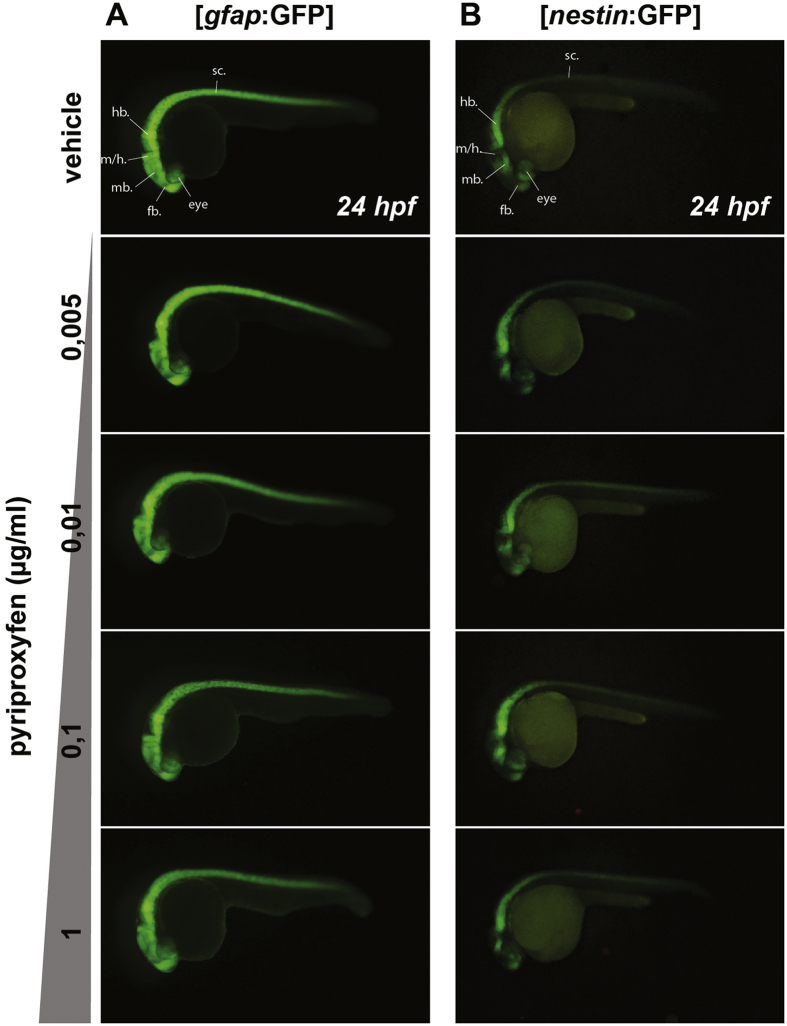
Neural stem cells regional expression pattern is not affected by pyriproxyfen exposure. 24 hpf transgenic embryos expressing the GFP under the gfap promoter [*gfap:*GFP] (**A**) or nestin promoter [*nestin:*GFP] (**B**) were treated with different doses of pyriproxyfen from the 2–4 cell stage onwards. Whole GFP expression pattern of both transgenic lines is not affected by the treatment. (N = 2, n = 25 per condition). sc.: spinal cord; hb.: hindbrain; m/h: midbrain/hindbrain boundary; mb.: midbrain; fb.: forebrain.

As several studies correlated a microcephaly phenotype with a depletion in the neural stem cell population and/or an alteration of their proliferation state^40^,^41^,^42^,^43^,^44^, we decided to investigate the effects of pyriproxyfen on the number of neural stem cells (NSCs) during neurodevelopment. To do so, we used two transgenic lines: [*nestin:*GFP] and [*gfap:*GFP] in which the main NSCs are fluorescently labeled, and exposed freshly laid eggs to increasing doses of pyriproxyfen from 0.005 μg/ml up to 1 μg/ml^45^,^46^. Firstly, we monitored the GFP expression pattern in whole embryos at 24 hours post fertilization (hpf), near the time of neurogenesis (Fig. 4). The *gfap* promoter drives strong GFP expression throughout the nervous system (Fig. 4A), whereas the *nestin* promoter drives a fainter expression more localized in the rostral regions of the nervous system (Fig. 4B). In both cases, we did not notice any difference in the regional expression pattern of GFP in either transgenic line following exposure to pyriproxyfen.

**Figure 5:**
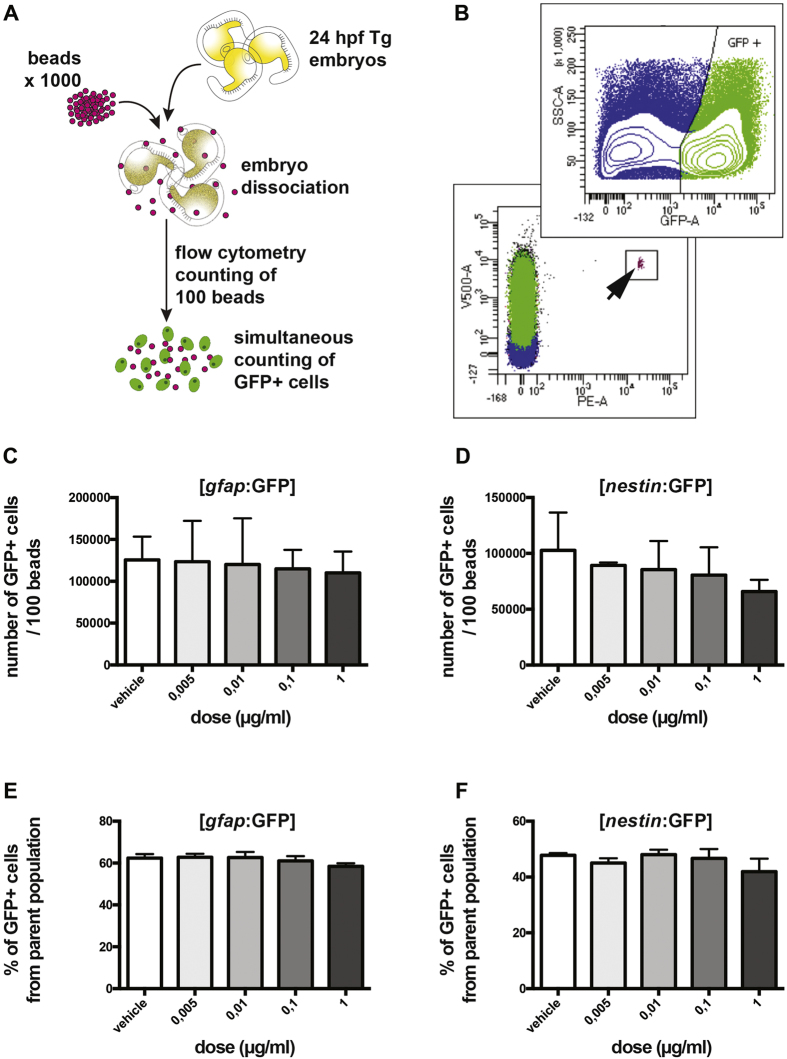
Neural stem cell populations are not affected by pyriproxyfen exposure. (**A**) Whole 24 hpf transgenic embryos ([*gfap:*GFP] or [*nestin:*GFP] were dissociated into single-cells in a medium containing a 1,000 fluorescent beads. 100 beads were counted by flow cytometry and the number of GFP+ cells was counted simultaneously. (**B**) The GFP+ cell population ([*gfap:*GFP] embryos) was distinguishable using a 488 nm laser with a 530/30 BP filter (upper panel). The fluorescent beads were distinguishable using a 561 nm laser with a 610/20 BP filter and a 405 nm laser with a 525/50 BP filter. The beads are completely separated from GFP+ cells on a FSC/SSC dot plot (lower panel, arrow). (**C**,**D**) The number of gfap+ cells and nestin+ counted per 100 beads was not significantly different upon pyriproxyfen exposure when compared to vehicle-treated embryos (*p* > 0.05). (**E**,**F**) The same results presented as a percentage of GFP+ cells/parent population also show no significant differences upon pyriproxyfen exposure among both gfap+ cells and nestin+ cells (*p* > 0.05). (N = 2, n = 25 per condition).

Although the results indicate that these early neural populations are not affected, we wanted to quantify more precisely this observation as GFP fluorescence itself is hard to quantify solely by imaging. To do so, we used flow cytometry of single-cells isolated from whole *in vivo* 24 hpf transgenic embryos (either [*gfap:*GFP] or [*nestin:*GFP]). Indeed, for each transgenic line, we dissociated 25 embryos that were previously exposed to various doses of pyriproxyfen beginning from the 2–4 cell stage, into single cells for further flow cytometry counting of GFP-positive cells (GFP+) (Fig. 5). To prevent any bias in the quantification of the exact number of GFP+ cells in each sample, we added 1,000 fluorescent beads among embryos prior to dissociation in order to normalize the final cell count. After dissociation, we counted an exact number of 100 beads by flow cytometry from the single-cell suspension (containing fluorescent beads) and assessed how many GFP+ cells were sorted simultaneously (Fig. 5A). Using different laser wavelengths (see methods), we were able to discriminate the GFP+ population (Fig. 5B, upper panel) as well as the fluorescent beads (Fig. 5B, lower panel). This normalization method avoids bias in the absolute quantification of cells that could occur during the experiment, as for example the loss of volume and cells during pipetting and tube transfers.

FACS-quantification using normalising beads has already been described (ref. 47). However, in order to ensure that our method would detect significantly any change in GFP+ cell numbers, we quantified nestin+ cells from 18 hpf and 24 hpf embryos (Supplemental Figure 4). At these early stages in development, the embryo is undergoing a major wave of neurogenesis and the number of stem cells is expected to increase drastically. As expected, we observed a significant increase with time in the number of nestin+ cells that have been quantified by our FACS method between these two stages. This confirms that our quantification method is accurate enough to distinguish a change in cell number.

Using this normalized method, we observed that the number of GFP+ cells counted simultaneously with the 100 beads (referred to as # of GFP+ cells/100 beads) was not significantly different among any of the concentrations of pyriproxyfen tested in the [*gfap:*GFP] (*p* > 0.05, one-way ANOVA, Dunnett’s multiple comparisons test, N = 2, n = 25; Fig. 5C) nor the [*nestin:*GFP] (*p* > 0.05, one-way ANOVA, Dunnett’s multiple comparisons test, N = 2, n = 25; Fig. 5D) transgenic embryos. We also analyzed these data as percentage of GFP+ cells compared to the total population of cells counted during the flow cytometry assay and found no significant difference between pyriproxyfen-exposed and vehicle-treated embryos in both the [*gfap:*GFP] (*p* > 0.05, one-way ANOVA, Dunnett’s multiple comparisons test, N = 2, n = 25; Fig. 5E) and [*nestin:*GFP] (*p* > 0.05, one-way ANOVA, Dunnett’s multiple comparisons test, N = 2, n = 25; Fig. 5F) transgenic embryos.

Altogether, these results suggest that pyriproxyfen exposure has no effect on the number, nor the whole regional pattern of the main early neural populations (gfap+, nestin+).

## Discussion

Pyriproxyfen has been recently brought into the public eye following an intense debate involving anecdotal claims that it may potentially be the cause of the surge of microcephaly cases reported in the Americas, as it is commonly used in drinking water as a mosquito growth-inhibitor. Although the effects of this compound on vertebrate metabolism and neurotoxicity have been reported^31^,^32^,^33^, no study to date has directly tested its effects specifically on the development of the nervous system. Therefore, in this work, we aimed to do so using zebrafish as a convenient toxicology model. Our results show that using [*elavl3:*GFP] transgenic fish and an algorithm for 3D confocal-based reconstruction (Imaris, Bitplane), whole brain volume development was not affected by pyriproxyfen in zebrafish embryos (2 dpf) or at later larval stages (7 dpf). Moreover, the main morphological structures within the brain were not affected even at concentrations 100-fold higher than the maximum dose of pyriproxyfen used in practice as recommended by the WHO^22^.

Furthermore, we sought to investigate the potentially more subtle effects of pyriproxyfen among two main neural stem cell populations that are important during neurodevelopment. Using [*nestin:*GFP] and [*gfap:*GFP] transgenic embryos, we were able to rigorously quantify the number of GFP+ cells (e.g NSCs) *in vivo* in 24 hpf embryos treated with increasing concentrations of pyriproxyfen. Although gfap and nestin populations correspond to different states of neurogenic differentiation steps in mammals^48^,^49^, the discrimination between these two subclasses of NSCs is not as clearly understood during neurogenesis in the zebrafish. Our work shows that amongst these two NSC populations, both considered as early, undifferentiated neural populations, neither is affected by pyriproxyfen exposure. Of note is that although gfap and nestin are canonical markers of NSCs, some recent studies discriminated different subclasses of NSCs^48^,^50^, also in zebrafish^51^. As a result, one cannot rule out that pyriproxyfen could have subtle effects on a subclass of NSCs that could not have been discriminated by looking at the gfap+ and nestin+ populations on their whole.

Although no developmental abnormalities are induced by doses of pyriproxyfen neighbouring the maximum concentration used in practice as recommended by the WHO, we did notice a strong teratogenic effect at extremely high doses exceeding 1 μg/ml. Indeed, zebrafish embryos treated with these extremely high doses from the earliest stages of development onwards do not survive after two days of development. As a result, we suggest that the use of pyriproxyfen should be monitored closely and that the search for other alternative methods should be sought after.

In summary, our results suggest that pyriproxyfen alone is unlikely to cause neurodevelopmental effects that could explain the rise of microcephaly cases that have been reported in the past several months. As a result, our work emphasizes the need to focus research efforts on unraveling the true source of these microcephaly cases, keeping in mind that it may involve a complex combination of causes. As the ZIKV infection is now becoming more and more strongly correlated with the emergence of these microcephaly phenotypes^42^,^52^,^53^,^54^, we recommend that the scientific community continue to pursue research along this path.

## Materials and Methods

### Fish Husbandry

Wild-type zebrafish (*Danio rerio*) were reared at 28.5 °C, kept under a 12 hr dark, 12 hr light cycle and staged as described previously^55^. They were bred according to standard procedures^56^. All experiments were performed in compliance with the guidelines of the Canadian Council for Animal Care and conducted at the Research Center of the University of Montreal Hospital Center (CRCHUM). All the experimental protocols were performed under the approval of the vertebrate animal welfare assurance from the Institutional Animal Care and Use Committee (IACUC) for the use of adult zebrafish (approved on 2015/08/31). The *elavl3, nestin* and *gfap* transgenic lines were previously generated as described^37^,^43^ and are routinely maintained in our fish facility at the CRCHUM.

### Pyriproxyfen treatment assay

Transgenic [*elavl3*:GFP] embryos were treated from 2–4 cell stage onwards with various pyriproxyfen (4-phenoxyphenyl (RS)-2-(2-pyridyloxy)propyl ether) (Sigma cat#34174) concentrations (μg/ml) from a stock solution of 100 g/L diluted in 100% ethanol (EtOH) or DMSO: untreated, vehicle, 0.005, 0.01, 0.05, 0.1, 0.35, 1, 10, 100. Pyriproxyfen was diluted into 50 ml of zebrafish system water according to desired final treatment concentration. The final concentration of EtOH or DMSO in the fish water was 0,1%. Furthermore, at 8 hpf, 1-phenyl 2-thiourea (PTU) (Sigma) was added to each vial at a final concentration of 0.003% in order to prevent pigmentation of the embryo, as previously described^57^, enabling brain volume analysis at 2 and 7 dpf. The medium was changed every 2 days in which fresh pyriproxyfen and PTU were added, over the course of 8 days. The final 50 ml volume contained 20 embryos for each condition. All incubations were performed in glass vials to prevent any sequestration of the compound by plastic.

### Liquid chromatography-mass spectrometry detection

LC-MS grade solvent, acetonitrile was obtained from EMD Millipore, formic acid from Fisher Scientific and MilliQ filtered water was used. The embryos were washed 3 times with clean aquarium water and 200 μL of the last washing solution was kept for further LC-MS analysis. The embryos (4 to 6 embryos) were extracted with 200 μL of acetonitrile. The mixture was vortexed (10 s) and sonicated (5 min) two times to obtain a white suspension. After 10 min of centrifugation (15000 rpm, 19 °C), 220 μL of the supernatant was transferred in a new 1.5 ml tube and dried down under vacuum for 45 min. The dry pellet was resuspended in 100 μL of acetonitrile and the different treated concentrations 0.01 μg/ml, 0.1 μg/ml and 1 μg/ml were diluted by 100, 1000 and 10000 respectively in acetonitrile/water 0.1% formic acid (1:1 v/v) prior to analysis. For the LC-MS/MS assay, analyses were collected in positive mode on an triple quadrupole MS system (EVOQ Elite, Bruker, Billerica, MA) coupled with an ultrahigh-performance liquid chromatography pump (Advance, Bruker) and equipped with a reversed-phase Poroshell 120EC-C18 (Agilent, 4.6 × 50 mm, 2.7 μm). Mobile phase phases were water with 0.1% formic acid (A) and acetonitrile with 0.1% formic acid (B). Pyriproxyfen was eluted at 3.64 min with a gradient from 30%B to 90% B in 3 min follow by 3 min at 90% and then, returned to initial conditions with an equilibration of 1 min. Column temperature was 45 °C, flow rate was 1 ml/min. The sample injection volume was set at 1 μL and repeated three times for each sample. The operating parameters of the mass spectrometer were: positive spray voltage 3500 V, cone temperature 350 °C, cone gas flow 20 (arbitrary units), heated probe temperature 500 °C, probe gas flow 40 (arbitrary units), nebulizer gas flow 60 (arbitrary units). Collision energy of pyriproxyfen was optimized from a continuous flow of a standard injection (300 nM at 5 μL/min). Two multiple reaction monitoring (MRM) transitions were acquired: 322 → 96 (14.0 V), 322 → 185 (22.0 V). Quantification of pyriproxyfen in embryos, based on peak areas, was performed by external calibration. Seven points calibration curve from 0.195 to 12.5 nM was generated using linear regression analysis and the linearity was qualified by linear correlation coefficient, R^2^.

### Survival and gross morphological analysis

The number of dead or deformed embryos or hatched larvae were counted for the following 10 pyriproxyfen treatment groups every day (μg/ml): untreated, EtOH, 0.005, 0.01, 0.05, 0.1, 0.35, 1, 10, 100. Survival was examined until 7 dpf. At 7 dpf, we also examined larval length and eye diameter to examine subtle developmental defects that may have arisen in our treatment groups.

### Brain volume imaging and analysis

For brain volume analysis, 2 and 7 dpf larvae were embedded in low melting point (LMP) agarose (Invitrogen) and were positioned dorsal up in order to facilitate imaging the dorsal aspect of the larvae. Brain volumes were visualized using a Quorum Technologies spinning disk confocal microscope with a CSU10B (Yokogawa) spinning head mounted on an Olympus BX61W1 fluorescence microscope and connected to a Hamamatsu ORCA-ER camera. Images were acquired using Volocity software (Improvision) and analyzed using Imaris software (Bitplane).

### Single-cell dissociation and FACS

A thousand 123count ebeads (Biosciences #01-1234-42) were added to embryos at 24 hours post-fertilization (hpf). They were briefly washed in calcium-free Ringer’s solution and de-yolked by up and down pipetting. De-yolked embryos were pelleted by 500 × g centrifugation for 5 min. They were briefly washed with FACSmax cell dissociation solution (Genlantis) and transferred in a 60 mm petri dish with FACSmax solution, then incubated at 28.5 °C. Single-cell dissociation was carefully monitored every 5 min and was generally achieved within 30 min of incubation. Efficient dissociation was helped by firmly tapping the petri dish and by gentle pipetting. Single cells were exhaustively washed twice in cold PBS, pelleted and resuspended in 500 μL of cold PBS. Single cells were filtered in a Falcon tube with a cell strainer cap (Fisher Scientific) and placed on ice until counting. Samples were analyzed using a LSRII flow cytometer with DIVA 8 software (BD Biosciences San Jose, CA, USA). GFP expressing cells were identified using a 488 nm laser and a 530/30 BP filter and 123count ebeads with a 561 laser and a 610/20 filter and a 405 laser with a 525/50 filter. 123count ebeads are completely separated from cells on a FSC/SSC dot plot.

### Statistical analysis

Graphpad 6.0 (Prism) was used to assess data groupings for significance. Statistical analyses used one-way unpaired ANOVA, followed by a *post hoc* Dunnett’s multiple comparisons test. For datasets with a non-normal distribution, assessed by performing a Shapiro-Wilk normality test, non-parametric tests were used where an unpaired Kruskal–Wallis test was performed, followed by Dunn’s *post hoc* test for multiple comparisons. Significance was assessed at *p* < 0.05. N is the number of datasets examined and n is the number of larvae used in each treatment group for within each N. Data in figures are presented as mean ± S.E.M.

## Additional Information

**How to cite this article**: Dzieciolowska, S. *et al*. The larvicide pyriproxyfen blamed during the Zika virus outbreak does not cause microcephaly in zebrafish embryos. *Sci. Rep.* **7**, 40067; doi: 10.1038/srep40067 (2017).

**Publisher's note:** Springer Nature remains neutral with regard to jurisdictional claims in published maps and institutional affiliations.

## Supplementary Material

Supplementary Information

## References

[b1] Lazear H. M., Stringer E. M. & de Silva A. M. The Emerging Zika Virus Epidemic in the Americas: Research Priorities. JAMA: the journal of the American Medical Association 315, 1945–1946, doi: 10.1001/jama.2016.2899 (2016). 26963564

[b2] Gatherer D. & Kohl A. Zika virus: a previously slow pandemic spreads rapidly through the Americas. J Gen Virol 97, 269–273, doi: 10.1099/jgv.0.000381 (2016). 26684466

[b3] Duffy M. R. . Zika virus outbreak on Yap Island, Federated States of Micronesia. The New England journal of medicine 360, 2536–2543, doi: 10.1056/NEJMoa0805715 (2009). 19516034

[b4] Oliveira Melo A. S. . Zika virus intrauterine infection causes fetal brain abnormality and microcephaly: tip of the iceberg? Ultrasound Obstet Gynecol 47, 6–7, doi: 10.1002/uog.15831 (2016). 26731034

[b5] Corona-Rivera J. R. . Report and review of the fetal brain disruption sequence. Eur J Pediatr 160, 664–667 (2001). 11760023 10.1007/s004310100813

[b6] Avery G. B., Meneses L. & Lodge A. The clinical significance of “measurement microcephaly”. Am J Dis Child 123, 214–217 (1972). 5026200 10.1001/archpedi.1972.02110090084008

[b7] Van den Eeden S. K., Karagas M. R., Daling J. R. & Vaughan T. L. A case-control study of maternal smoking and congenital malformations. Paediatr Perinat Epidemiol 4, 147–155 (1990). 2362871 10.1111/j.1365-3016.1990.tb00630.x

[b8] Ouellette E. M., Rosett H. L., Rosman N. P. & Weiner L. Adverse effects on offspring of maternal alcohol abuse during pregnancy. The New England journal of medicine 297, 528–530, doi: 10.1056/NEJM197709082971003 (1977). 887104

[b9] Samson H. H. & Grant K. A. Ethanol-induced microcephaly in neonatal rats: relation to dose. Alcohol Clin Exp Res 8, 201–203 (1984). 6375432 10.1111/j.1530-0277.1984.tb05839.x

[b10] Superti-Furga A., Steinmann B., Duc G. & Gitzelmann R. Microcephaly and maternal phenylketonuria. Eur J Pediatr 155, 992 (1996). 8911906 10.1007/BF02282896

[b11] Rouse B. . Maternal phenylketonuria syndrome: congenital heart defects, microcephaly, and developmental outcomes. The Journal of pediatrics 136, 57–61 (2000). 10636975 10.1016/s0022-3476(00)90050-7

[b12] Cowie V. A. Microcephaly: a review of genetic implications in its causation. J Ment Defic Res 31 (Pt 3), 229–233 (1987). 3316660 10.1111/j.1365-2788.1987.tb01365.x

[b13] Marcondes C. B. & Ximenes Mde F. Zika virus in Brazil and the danger of infestation by Aedes (Stegomyia) mosquitoes. Rev Soc Bras Med Trop 49, 4–10, doi: 10.1590/0037-8682-0220-2015 (2016). 26689277

[b14] (ECDC) European Centre for Disease Prevention and Control. *Zika virus epidemic in the Americas: potential association with microcephaly and Guillain-Barré syndrome*, http://ecdc.europa.eu/en/publications/publications/zika-virus-americas-association-with-microcephaly-rapid-risk-assessment.pdf (2015).

[b15] Ministério da Saúde (Brazil). *Ministério divulga boletim epidemiológico sobre microcefalia*, http://portalsaude.saude.gov.br/index.php/o-ministerio/principal/secretarias/svs/noticias-svs/20929-ministerio-divulga-boletim-epidemiologico-sobre-microcefalia (2015) (20/08/2016).

[b16] Mlakar J. . Zika Virus Associated with Microcephaly. The New England journal of medicine 374, 951–958, doi: 10.1056/NEJMoa1600651 (2016). 26862926

[b17] Schuler-Faccini L. . Possible Association Between Zika Virus Infection and Microcephaly - Brazil, 2015. MMWR Morb Mortal Wkly Rep 65, 59–62, doi: 10.15585/mmwr.mm6503e2 (2016). 26820244

[b18] Wheeler D. E. & Nijhout H. F. A perspective for understanding the modes of juvenile hormone action as a lipid signaling system. Bioessays 25, 994–1001, doi: 10.1002/bies.10337 (2003). 14505366

[b19] Riddiford L. M. Juvenile hormone action: a 2007 perspective. J Insect Physiol 54, 895–901, doi: 10.1016/j.jinsphys.2008.01.014 (2008). 18355835

[b20] Wilson T. G. The molecular site of action of juvenile hormone and juvenile hormone insecticides during metamorphosis: how these compounds kill insects. J Insect Physiol 50, 111–121, doi: 10.1016/j.jinsphys.2003.12.004 (2004). 15019512

[b21] Riddiford L. M. Prevention of metamorphosis by exposure of insect eggs to juvenile hormone analogs. Science 167, 287–288, doi: 10.1126/science.167.3916.287 (1970). 17734449

[b22] (WHO) World Health Organization. *Pyriproxyfen in Drinking-water: Use for Vector Control in Drinking-water Sources and Containers* http://www.who.int/water_sanitation_health/dwq/chemicals/pyriproxyfenvector.pdf (2007) (25/08/2016).

[b23] (WHOPES) World Health Organization Pesticide Evaluation Scheme. *WHOPES-recommended compounds and formulations for control of mosquito larvae* http://www.who.int/whopes/quality/newspecif/en/ (2012) (20/08/2016).

[b24] (REDUAS) Red Universitaria de Ambiente Y Salud. *REPORT from Physicians in the Crop-Sprayed Town regarding Dengue-Zika, microcephaly, and massive spraying with chemical poisons*, http://reduas.com.ar/report-from-physicians-in-the-crop-sprayed-town-regarding-dengue-zika-microcephaly-and-massive-spraying-with-chemical-poisons/ (25/08/2016).

[b25] (ABRASCO) Brazillian Association of Collective Health. Nota técnica sobre microcefalia e doenças vetoriais relacionadas ao Aedes aegypti: os perigos das abordagens com larvicidas e nebulizações químicas – fumacê, https://www.abrasco.org.br/site/2016/02/nota-tecnica-sobre-microcefalia-e-doencas-vetoriais-relacionadas-ao-aedes-aegypti-os-perigos-das-abordagens-com-larvicidas-e-nebulizacoes-quimicas-fumace/ (25/08/2016).

[b26] Dwilson S. D. *Pyriproxyfen: 5 Fast Facts You Need to Know*, http://heavy.com/news/2016/02/pyriproxyfen-microcephaly-products-larvicide-zika-birth-defects-in-drinking-water-pet-flea-tick-treatments-piriproxifen/ (2016).

[b27] Freeman M. *Zika Or Insecticide Pyriproxyfen Behind Microcephaly Cases?*, http://www.activistpost.com/2016/02/zika-or-insecticide-pyriproxyfen-behind-microcephaly-cases.html (19/08/2016).

[b28] Russo S. *The Larvicide Affair: The Ripple Effects of Zika Misinformation in Brazil*, http://blogs.plos.org/scied/2016/03/31/zika-v-pesticides/ (19/08/2016).

[b29] FOX News Latino (FOX). *Brazilian state suspends larvicide used to combat Zika virus*, http://latino.foxnews.com/latino/news/2016/02/14/brazilian-state-suspends-larvicide-used-to-combat-zika-virus/ (20/08/2016).

[b30] Almendrala A. *A Viral Story Links The Zika Crisis To Monsanto*. *Don*’*t Believe It*., http://www.huffingtonpost.com/entry/zika-monsanto-pyriproxyfen-microcephaly_us_56c2712de4b0b40245c79f7c?utm_hp_ref=tw (19/08/2016).

[b31] (FAO) Food and Agriculture Organization of the United Nations. *PYRIPROXYFEN*, http://www.fao.org/fileadmin/templates/agphome/documents/Pests_Pesticides/JMPR/Evaluation99/25Pyriproxyfen.pdf (1999) (25/08/2016).

[b32] (FAO) Food and Agriculture Organization of the United Nations. PYRIPROXYFEN. http://www.fao.org/fileadmin/templates/agphome/documents/Pests_Pesticides/JMPR/Reports_1991-2006/REPORT1999.pdf (1999) (25/08/2016).

[b33] Koyama Y. . [A six-month chronic dietary toxicity study of pyriproxyfen in rats]. J Toxicol Sci 14, 43–64 (1989). 2738965 10.2131/jts.14.43

[b34] Nishimura Y. . Zebrafish as a systems toxicology model for developmental neurotoxicity testing. Congenital anomalies 55, 1–16 (2015). 25109898 10.1111/cga.12079

[b35] Braunbeck T. . The fish embryo test (FET): origin, applications, and future. Environ Sci Pollut Res Int 22, 16247–16261, doi: 10.1007/s11356-014-3814-7 (2015). 25395325

[b36] OECD. Oecd Guidelines For The Testing Of Chemicals: Fish Embryo Acute Toxicity (FET) Test. http://www.oecd-ilibrary.org/docserver/download/9713161e.pdf?expires=1480004944&id=id&accname=guest&checksum=8020D61E1152DD9A9E8347B7-EFC88E50 (2013) (18/08/2016).

[b37] Truong L., Gonnerman G., Simonich M. T. & Tanguay R. L. Assessment of the developmental and neurotoxicity of the mosquito control larvicide, pyriproxyfen, using embryonic zebrafish. Environ Pollut 218, 1089–1093, doi: 10.1016/j.envpol.2016.08.061 (2016). 27593350 PMC5048575

[b38] Park H. C. . Analysis of upstream elements in the HuC promoter leads to the establishment of transgenic zebrafish with fluorescent neurons. Dev Biol 227, 279–293, doi: 10.1006/dbio.2000.9898 (2000). 11071755

[b39] Capo-Chichi J. M. . Disruption of CLPB is associated with congenital microcephaly, severe encephalopathy and 3-methylglutaconic aciduria. J Med Genet 52, 303–311, doi: 10.1136/jmedgenet-2014-102952 (2015). 25650066

[b40] Li H. . Zika Virus Infects Neural Progenitors in the Adult Mouse Brain and Alters Proliferation. Cell Stem Cell, doi: 10.1016/j.stem.2016.08.005 (2016). PMC5097023 27545505

[b41] Nguyen H. N., Qian X., Song H. & Ming G. L. Neural stem cells attacked by Zika virus. Cell Res 26, 753–754, doi: 10.1038/cr.2016.68 (2016). 27283801 PMC5129882

[b42] Li C. . Zika Virus Disrupts Neural Progenitor Development and Leads to Microcephaly in Mice. Cell Stem Cell 19, 120–126, doi: 10.1016/j.stem.2016.04.017 (2016). 27179424

[b43] Dang J. . Zika Virus Depletes Neural Progenitors in Human Cerebral Organoids through Activation of the Innate Immune Receptor TLR3. Cell Stem Cell 19, 258–265, doi: 10.1016/j.stem.2016.04.014 (2016). 27162029 PMC5116380

[b44] Tang H. . Zika Virus Infects Human Cortical Neural Progenitors and Attenuates Their Growth. Cell Stem Cell 18, 587–590, doi: 10.1016/j.stem.2016.02.016 (2016). 26952870 PMC5299540

[b45] Lam C. S., Marz M. & Strahle U. gfap and nestin reporter lines reveal characteristics of neural progenitors in the adult zebrafish brain. Dev Dyn 238, 475–486, doi: 10.1002/dvdy.21853 (2009). 19161226

[b46] Bernardos R. L. & Raymond P. A. GFAP transgenic zebrafish. Gene Expr Patterns 6, 1007–1013, doi: 10.1016/j.modgep.2006.04.006 (2006). 16765104

[b47] Yuan X., Wu Q., Li H., Li B. & Xiu R. Absolute circulating pericyte progenitor cell counts in mice by flow cytometry: comparison of 2 single-platform technologies. Int J Biol Markers 30, e434–438, doi: 10.5301/jbm.5000156 (2015). 26109365

[b48] Codega P. . Prospective identification and purification of quiescent adult neural stem cells from their *in vivo* niche. Neuron 82, 545–559, doi: 10.1016/j.neuron.2014.02.039 (2014). 24811379 PMC4360885

[b49] Wang Y. Z., Plane J. M., Jiang P., Zhou C. J. & Deng W. Concise review: Quiescent and active states of endogenous adult neural stem cells: identification and characterization. Stem Cells 29, 907–912, doi: 10.1002/stem.644 (2011). 21557389 PMC3306660

[b50] Yabut O. & Pleasure S. J. The quintessence of quiescence. Neuron 82, 501–503, doi: 10.1016/j.neuron.2014.04.025 (2014). 24811373

[b51] Johnson K. . Gfap-positive radial glial cells are an essential progenitor population for later-born neurons and glia in the zebrafish spinal cord. Glia 64, 1170–1189, doi: 10.1002/glia.22990 (2016). 27100776 PMC4918407

[b52] Tang B. L. Zika virus as a causative agent for primary microencephaly: the evidence so far. Arch Microbiol 198, 595–601, doi: 10.1007/s00203-016-1268-7 (2016). 27412681

[b53] Valentine G., Marquez L. & Pammi M. Zika Virus-Associated Microcephaly and Eye Lesions in the Newborn. J Pediatric Infect Dis Soc 5, 323–328, doi: 10.1093/jpids/piw037 (2016). 27405738

[b54] Araujo A. Q., Silva M. T. & Araujo A. P. Zika virus-associated neurological disorders: a review. Brain 139, 2122–2130, doi: 10.1093/brain/aww158 (2016). 27357348

[b55] Kimmel C. B., Ballard W. W., Kimmel S. R., Ullmann B. & Schilling T. F. Stages of embryonic development of the zebrafish. Dev Dyn 203, 253–310, doi: 10.1002/aja.1002030302 (1995). 8589427

[b56] Westerfield M. The Zebrafish Book: A Guide for the Laboratory Use of Zebrafish (Danio rerio) (Institute of Neuro Science, 1995).

[b57] Karlsson J., von Hofsten J. & Olsson P. E. Generating transparent zebrafish: a refined method to improve detection of gene expression during embryonic development. Mar Biotechnol (NY) 3, 522–527, doi: 10.1007/s1012601-0053-4 (2001). 14961324

