# Supplementary material for: The larvicide pyriproxyfen blamed during the Zika virus outbreak does not cause microcephaly in zebrafish embryos
Source: Sci Rep. 2017 Jan 4;7:40067. doi: 10.1038/srep40067 (PMC5209666; doi:10.1038/srep40067)
Supplement: Supplementary Information [file srep40067-s1.pdf]

Supplementary information

Manuscript SREP-16-36307: ***The larvicide pyriproxyfen blamed during the Zika virus outbreak does not cause microcephaly in zebrafish embryos.***

By Stefania Dzieciolowska, Anne-Laure Larroque, Elizabeth-Ann Kranjec, Pierre Drapeau and Eric Samarut

Supplementary Figures

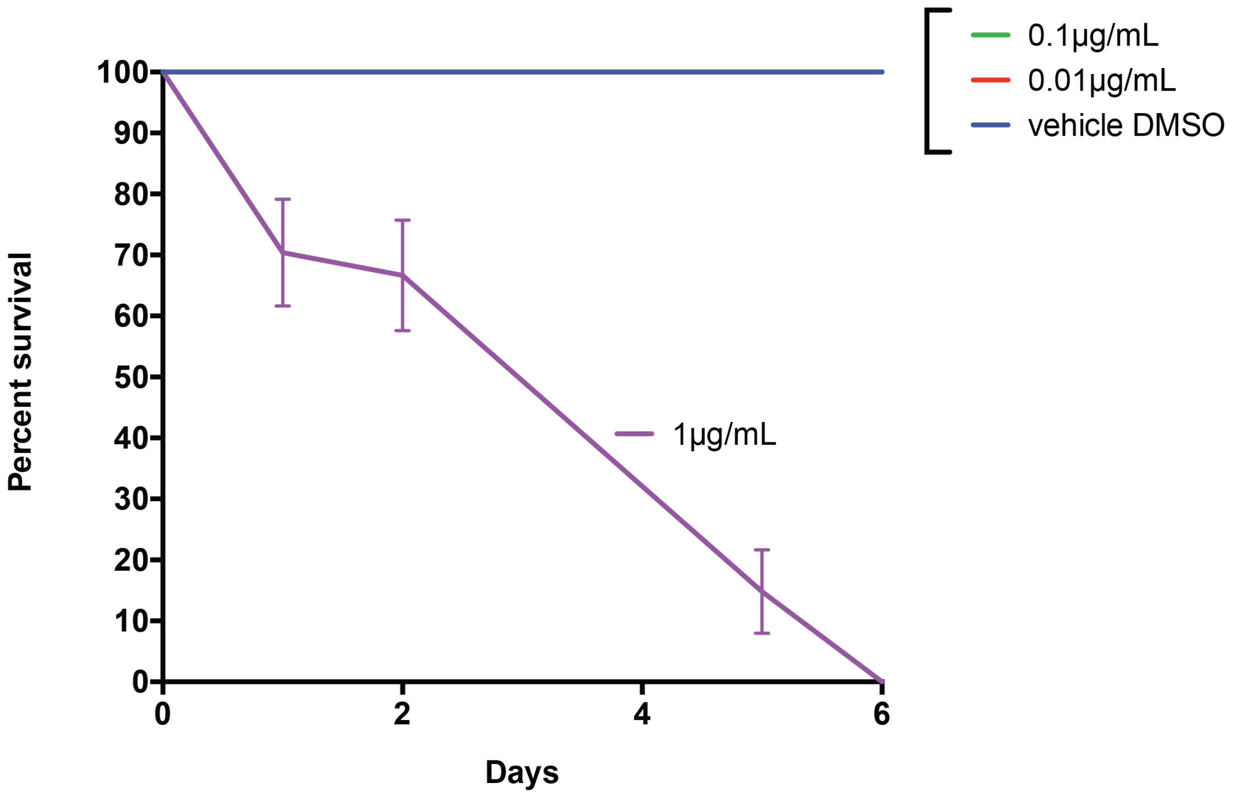

Dzieciolowska et al., Supp. Figure 1

A

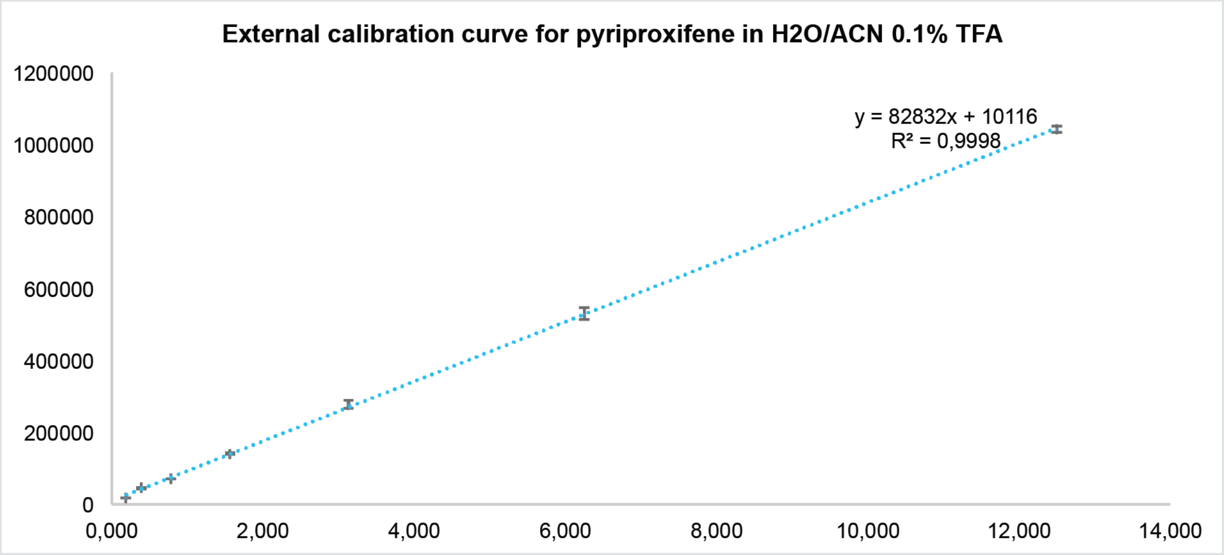

B

Pyriproxyfen amounts contained in zebrafish embryos following different water concentration exposition						
Pyriproxyfen water concentration (µg/mL)	Number of embryos in water	Dilution factor applied before sample analysis	Area response (a.u.)	Amounts per embryo (ng/embryo)	**Mean amount per embryo (ng/embryo)**	Std. dev. (ng/embryo)
1	4	1/10000	128722	115,04243	**108**	7
			114620	101,36010		
			121543	108,07673		
0,1	6	1/1000	264943	16,47771	**17,3**	0,7
			284308	17,72998		
			285431	17,80283		
0,01	6	1/100	344778	2,16389	**2,14**	0,03
			335132	2,10165		
			343940	2,15854		

Dzieciolowska et al., Supplementary Figure 2

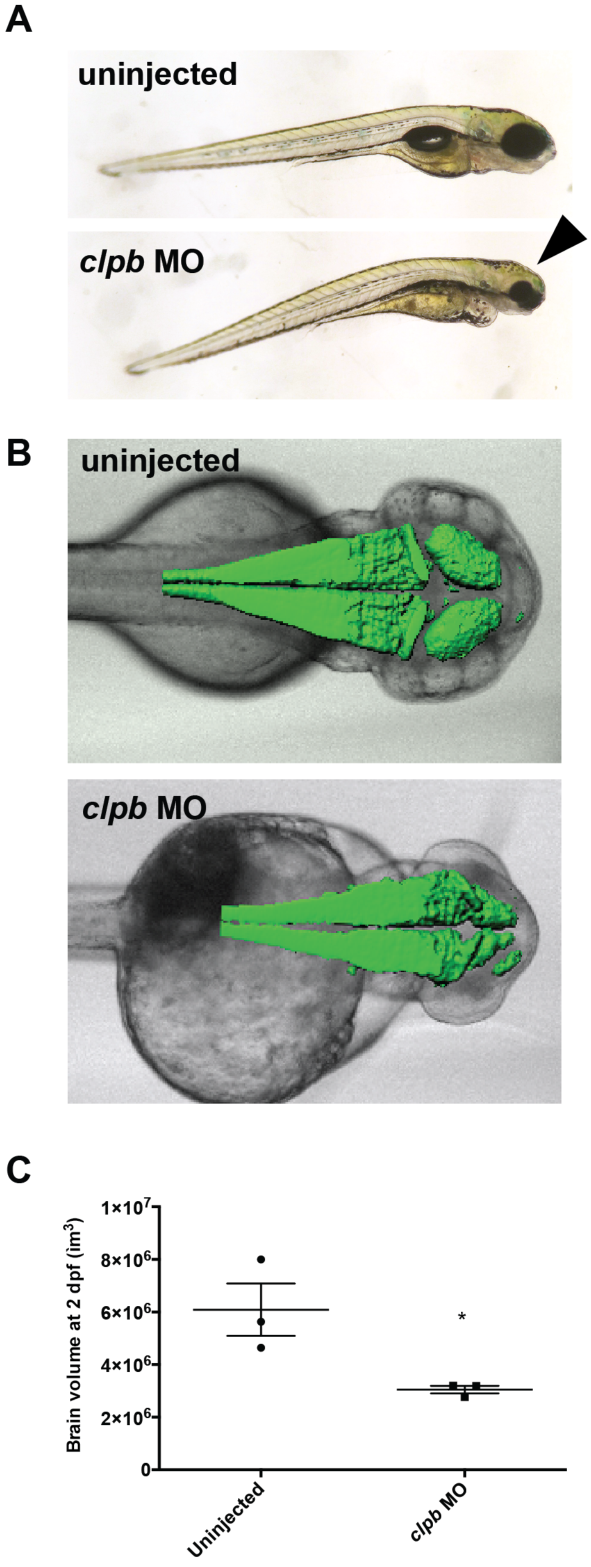

Dzieciolowska et al., Supp. Figure 3

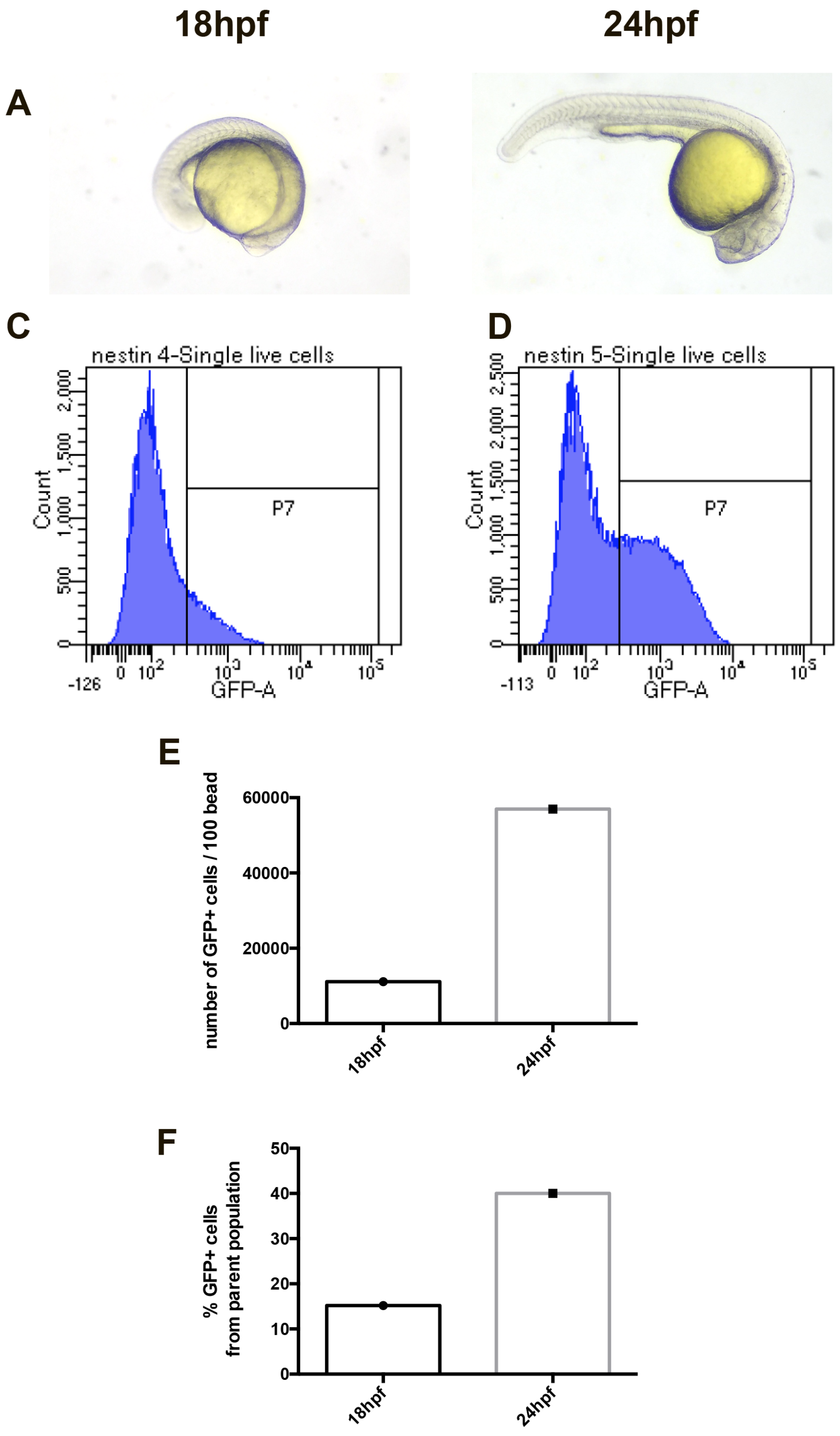

Dzieciolowska et al., Supplemental Figure 4

Legends to Supplementary figures

Supplementary Figure 1: Pyriproxyfen has the same effect when diluted into DMSO. Percentage of survival of embryos treated from 2-4 cell stage with pyriproxyfen concentrations of 0.01 µg/ml 0.1 µg/ml and 1 µg/ml. As observed in Figure 1, the latter dose appears to be lethal with 100% of death after 6 days of development. Lower doses of pyriproxyfen (0.01; 0.1 µg/ml) do not induce a severe decrease in the survival rate.

Supplementary Figure 2: LC-MS: Pyriproxyfen quantification (A) External calibration curve of pyriproxyfen. The equation of the curve is $y = 82832x + 10116$, where y represents area of response and x is the concentration in nM. The linear correlation coefficient (R^2) is 0.9998. (B) Quantification of pyriproxyfen per zebrafish embryo following the exposure to different concentrations in water.

Supplementary Figure 3: *clpb*-morpholino induced microcephaly leads to a reduced brain volume in zebrafish embryo. (A) 48 hpf embryos injected with a *clpb* morpholino exhibit a microcephaly phenotype (e.g reduced head size, black arrow head) as described by Capo-Chichi et al.[39] (B) elavl3:GFP embryos were injected with *clpb* morpholino and imaged at 48 hpf under a confocal microscope. (C) The 3D-brain volume calculation shows a reduced brain volume compared to uninjected embryos.

Supplementary Figure 4: nestin+ population quantification from 18 hpf versus 24 hpf embryos. (A) Bright field image of 18 and 24 hpf embryo. (B, C) The GFP+ cell population (e.g.[*nestin:*GFP]) was distinguishable using a 488 nm laser with a 530/30 BP

filter (population labeled P7). The fluorescent beads were distinguishable using a 561 nm laser with a 610/20 BP filter and a 405 nm laser with a 525/50 BP filter. (E) The number of nestin+ cells counted per 100 beads increases by a factor of 3 in 24 hpf embryos compared to 18 hpf embryos (F) The same results presented as a percentage of GFP+ cells/parent population also show an increase in the number of GFP+ cells at 24 hpf. (N=1, n=25 embryos)
